# Amazonian Shuar testosterone levels are related to parasite infection: immunocompetence handicap hypothesis implications

**DOI:** 10.1093/emph/eoag013

**Published:** 2026-06-28

**Authors:** Theresa E Gildner, Aaron D Blackwell, Melissa A Liebert, Samuel S Urlacher, Tara J Cepon-Robins, Joshua M Schrock, Richard G Bribiescas, Felicia C Madimenos, J Josh Snodgrass, Lawrence S Sugiyama

**Affiliations:** Department of Anthropology, Washington University in St. Louis, St. Louis, MI 63130, USA; Department of Anthropology, Washington State University, Pullman, WA 99164, USA; Department of Anthropology, Northern Arizona University, Flagstaff, AZ 86001, USA; Department of Anthropology, Baylor University, Waco, TX 76706, USA; Department of Anthropology, University of Colorado, Colorado Springs, CO, 80918, USA; Impact Institute, Northwestern University, Chicago, IL 60611, USA; Department of Anthropology, Yale University, New Haven, CT 06520, USA; Department of Anthropology, Queens College (CUNY), Flushing, New York, NY 11367, USA; Department of Anthropology, University of Oregon, Eugene, OR 97401, USA; Department of Anthropology, University of Oregon, Eugene, OR 97401, USA

**Keywords:** life history theory, evolutionary medicine, immunosuppression, soil-transmitted helminths, *Ascaris lumbricoides*, *Trichuris trichiura*

## Abstract

**Background:**

Testosterone is hypothesized to influence energetic tradeoffs between male reproduction and immune function. Testosterone is generally thought to be immunosuppressive, with high testosterone levels signaling male health and infection resistance. The Immunocompetence Handicap Hypothesis (ICHH) posits that testosterone’s role in developing male secondary sexual characteristics and suppressing immune function creates a tradeoff between testosterone-linked traits and immunocompetence. Yet, the immune effects of testosterone in humans remain poorly understood. Research is particularly limited among energetically constrained, natural fertility populations characterized by high pathogen loads and long-lasting infections (such as parasitic disease), characteristics relevant to the evolution of hypothesized testosterone-linked tradeoffs.

**Methods:**

We test whether salivary testosterone concentrations are associated with parasitic helminth infection status and parasite load among 90 Indigenous Shuar males aged 12–67 years.

**Results:**

Higher testosterone levels were associated with increased infection risk but lower parasite load. However, these findings were only evident among adolescents (ages 12–19) and only for *Trichuris trichiura* (whipworm) but not *Ascaris lumbricoides* (roundworm) infections.

**Conclusions:**

These findings suggest higher testosterone may increase the risk of becoming infected with some parasite species, but that heavy pathogen loads lead to a shift in resource allocation from testosterone production to immune activity during a life stage with high testosterone-related energetic demands. While some results aligned with the ICHH, the significant effects of participant age and parasite species on testosterone-infection associations indicate that the ICHH does not completely capture the full complexity of human immune-related life history tradeoffs and variation in host–parasite interactions.

## BACKGROUND

Life history theory predicts that because energetic resources are constrained, organisms face tradeoffs in their resource allocations among competing biological functions across the lifespan [1, 2]. Selection therefore produces context-dependent biological processes (including neuroendocrine signals) to regulate energy allocation in response to these tradeoffs. Testosterone plays a key role in male reproductive function [3, 4]. In humans, testosterone is low during childhood, rises during adolescence, peaks in young adulthood, and may decline during aging, directly affecting the development and maintenance of male-specific characteristics (e.g. secondary sexual characteristics such as muscle mass) throughout the life course [3, 4]. Energetically expensive testosterone-linked traits have been hypothesized to provide costly signals of a male’s genetic disease resistance that females use in mate choice, thus influencing male reproductive success and leading to selection for these male characteristics [5]. Testosterone may impact immune activity by binding to androgen receptors on immune cells. Several animal model studies link testosterone to poor health, including reduced white blood cell count and suppressed antibody production [6–9]. Consequently, researchers have hypothesized that testosterone impacts tradeoffs between reproductive effort and immunity across species [9–11].

One such hypothesis, the Immunocompetence Handicap Hypothesis (ICHH), posits that while testosterone plays a key role in the development and maintenance of male secondary sexual characteristics, it is also immunosuppressive, resulting in direct tradeoffs between immunocompetence and the development and maintenance of testosterone-linked traits [12]. However, this pattern is subject to change over the course of an infection, such that testosterone concentration is predicted to decrease as pathogen load increases, supporting greater investment in immune function. Once the infection is cleared, individual testosterone levels should rise, marking increased investment in mating effort [12]. High circulating testosterone levels are thus hypothesized to indicate low current pathogen load. While several studies across a range of species (e.g. birds, reptiles, fish) have found support for the ICHH [13–15], other non-human animal studies have produced insignificant results or findings that contradict the ICHH (e.g. higher testosterone levels linked with heavier infections) [16–18]. A common critique of the ICHH is that it is a relatively simplistic model. The impact of testosterone on immune activity may be shaped by several factors, including mating system (e.g. the importance of dominance rank to reproductive success), individual energetic status, and individual energetic condition (e.g. ability to support energetically costly aspects of both testosterone activity and immune function) [17].

To date, few studies have examined relationships between testosterone and immune activity in natural fertility, subsistence-based human populations who endure high infectious disease burdens—conditions that are most relevant to the evolution of hypothesized testosterone-linked tradeoffs [19]. This context is particularly relevant because energetic tradeoffs between distinct biological functions are expected to be more evident when demands on immune activity are high, but fewer energetic resources are available to support competing costs [20]. The limited available evidence suggests that the effects of testosterone on human immune activity may vary by immune response type. For instance, *ex vivo* antigen stimulation in whole blood samples collected from Indigenous Tsimane of Bolivia found that higher testosterone levels downregulated more costly aspects of immune function (e.g. T-cell mitogen induced cytokine production), but had no relationship with less costly forms of immune activation (e.g. B-cell induced cytokine production) [21]. Conversely, another study conducted among the Tsimane found that higher testosterone levels were positively associated with salivary mucosal immunity (measured using secretory IgA) [22]; a finding that aligns with an earlier study conducted among Filipino adult males [23]. This positive association may indicate increased immune investment at common pathogen entry points into the body, possibly to compensate for testosterone-linked suppression of other immune responses or to offset increased infection risk posed by intrasexual physical competition [22]. Together, these findings suggest that testosterone may be best described as immunoregulatory, rather than uniformly immunosuppressive [21, 24, 25].

## OBJECTIVES

More research is needed to clarify these complex patterns in humans by considering measures of both infection risk and intensity (i.e. infection status and pathogen load), particularly for persistent, long-lasting infections such as those caused by parasitic organisms like helminths (i.e. parasitic worms) [26]. Given the long coevolutionary history humans share with parasites and the costs of related immune defense, parasitic disease may have particularly important implications for evolved tradeoffs [27, 28]. It is also necessary to consider how the relative value of investment in testosterone-related traits and mating effort varies across the lifespan [9, 29]. Tradeoffs with immunity may be particularly acute when investments in male mating effort (and related traits) are highest, straining available resources. For instance, these tradeoffs may be especially evident during adolescence, a costly period of growth during which steroid hormone concentrations are shifting rapidly.

The present study addresses these outstanding questions by testing hypothesized tradeoffs between testosterone levels and parasite infection patterns among the Shuar—an Indigenous Ecuadorian population living in a high-pathogen context. Previous work indicates that parasite infection rates are relatively high among the Shuar, including soil-transmitted helminths (STH; parasitic worms spread through fecal-contaminated soil) such as *Ascaris lumbricoides* (large roundworm) and *Trichuris trichiura* (whipworm) [30–32]. Drawing on the ICHH, the following predictions are tested:

- **Prediction 1a: Higher testosterone concentrations will be associated with higher odds of parasite infection.** High testosterone levels are expected to be immunosuppressive [12]. Thus, risk of being infected is expected to be elevated in males with higher testosterone levels.
- **Prediction 1b: Higher testosterone concentrations during adolescence will be associated with higher odds of infection.** As documented in other studies [33, 34]—and given the energetic demands and changes in steroid hormone concentrations during this developmental period—we expect that higher testosterone levels among adolescents will be linked with increased rates of parasite infection.
- **Prediction 2: Lower testosterone concentrations will be associated with higher parasite loads.** Testosterone levels are expected to be downregulated during periods of immune activation triggered by higher intensity infections [12, 24, 35], so should be negatively related to parasite load (a proxy for infection intensity).

## METHODS

### Participants

This study was conducted as part of the Shuar Health and Life History Project (https://www.shuarproject.org/). The Shuar are an Indigenous population primarily living in the neotropical lowlands and Andean foothills of southeastern Ecuador and northeastern Peru [36, 37]. According to 2022 census data, ~110 000 Shuar live in Ecuador, primarily in the Morona-Santiago and Zamora Provinces [38]. Traditionally, Shuar lived in scattered households reliant on horticulture, fishing, hunting, and foraging [37, 39]. Shuar are currently engaging in rapid yet uneven market integration (i.e. the degree to which individuals produce for and consume from the market economy) [40], with this variation in turn influencing various health outcomes (including growth patterns and chronic disease risk) [40, 41]. Still, previous research indicates that parasitic infection is common among Shuar across various levels of market integration, with ~63% of 620 participants living in small rural and remote communities infected with at least one species of STH [30–32].

### Study design and sampling

This study employed a cross-sectional approach, with data collected over five field seasons in 11 Shuar communities (in the summers of 2012–14 and 2016–17). Random sampling was not feasible across this large population thus, in consultation with knowledgeable Shuar informants and political leaders—particularly the *Dirigentes de Salud* (health directors) of the *Federación Interprovincial de Centros Shuar*—a sample of communities were chosen based on their size, location, and interest in collaborating on this research. The sample included 90 males between the ages of 12–67 years old from 11 communities. The lower cutoff age of 12 was selected to capture those experiencing puberty at a younger age [42], to capture the full range of testosterone profiles across key developmental reproductive transitions. The median age at which Shuar males reach adult height is 20 years [42], so male adolescence was defined as 12–20 years old, consistent with cutoffs established by Bogin [43]. Informed verbal consent was obtained for all adult participants, with parental informed verbal consent and child informed assent obtained for all participants under 14 (the local age of consent). The study was approved by the Institutional Review Board of the University of Oregon and authorized by the *Federación Interprovincial de Centros Shuar*.

### Field and laboratory procedures

#### Salivary testosterone collection and analysis

Testosterone levels were measured using saliva samples, which provide useful measures of biologically active testosterone concentrations [44]. Saliva samples were collected via passive drool, wherein participants pooled saliva in their mouth and then gently ‘drooled’ directly into a 2.0 ml vial [45]. Due to diurnal fluctuation (daily changes in testosterone levels), at least 1 ml saliva was collected both before 9 am and after 4 pm for 3 consecutive days to establish individual diurnal patterns (a maximum of six samples per participant). Samples were collected directly by researchers, to ensure accurate timing of collection. Samples were stored in a portable −20°C field freezer until transfer to Quito, where they were shipped on dry ice via courier service to the Global Health Biomarker Lab at the University of Oregon for analysis. One researcher (TEG) analysed the samples using commercially available enzyme immunoassay kits from Salimetrics (Kit #1–2402; State College, PA), which have been well-validated to quantitatively assess salivary testosterone. Interassay coefficients of variation were 8.4 and 20.8% for high and low control samples, respectively, resulting in an overall interassay coefficient of 14.6%. The intraassay coefficient of variation was 8.4%. Analysis indicated that 1.7% of testosterone concentrations were below the lower limit of detection. These samples were excluded from analysis.

#### Stool collection and analysis

To determine parasite infection patterns, the first stool passage of the day was collected using previously described methods [30]. A single Kato-Katz smear was prepared from each participant’s fecal sample (by TEG or TJC) within an hour of sample collection [46]. Previous work indicates that the Kato-Katz technique accurately detects STH infections, especially in areas with high-intensity infections, with sensitivity estimates of over 94% for *A. lumbricoides* and *T. trichiura* from a single stool sample [47–49]. After ~30–60 minutes, the smears were examined using 10× and 40× microscopy by trained observers (either TEG or TJC) and species-specific EPG of feces recorded. Infection prevalence was calculated based on the proportion of participants with helminth ova in their stool.

#### Confounds

Variables known to influence testosterone levels or parasite infection patterns were controlled for during analysis. Ages were determined by self-report and birthdates on government issued identification cards, and crosschecked by informants and existing genealogical data, as previously described elsewhere [40]. Individual body mass index (BMI; kg/m^2^) has also been shown to influence immune function [50] and for adolescents height may indicate developmental pace. BMI was calculated based on height measured using a stadiometer (Seca Corporation 214, Hanover, MD) and weight measured using a Tanita scale (model BF680W). Recent anti-helminth medication use (reported by 2% of participants) did not affect model results and was excluded from analysis. We also tested models with coinfection variables (i.e. *T. trichiura* infection status was included as a covariate in the *A. lumbricoides* models, and vice versa), but the parameter estimates clustered around zero and did not affect other parameter estimates. The coinfection variables were therefore dropped from the final models.

### Statistical analyses

**Figure 1:**
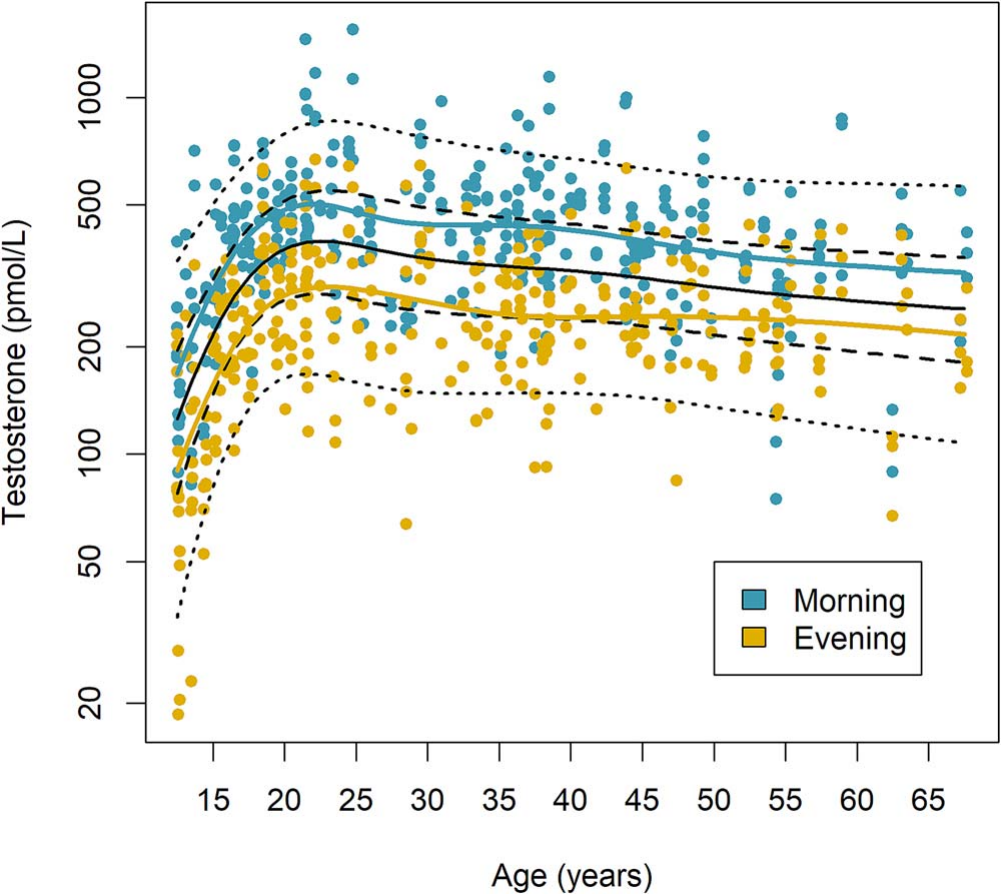
Testosterone by age. Black lines show the 5th, 25th, 50th, 75th, and 95th centiles from the GAMLSS model used to create age corrected z-scores. Blue and yellow colors show morning and evening testosterone concentrations, respectively. Median log scale morning and evening testosterone (colored lines) roughly follow the 25th and 75th centile lines, showing they are consistently ~1 SD apart, except in older ages where they narrow somewhat.

Analyses were conducted in R 4.2.1. All participants provided a stool sample for parasite infection analysis and at least three saliva samples; on average, 5.25 saliva samples were collected per participant. Height and weight were missing for 10 individuals; we, therefore, used random forest imputation with the *mice* package [51] to generate 10 datasets with imputed values for height and weight, from which BMI was calculated. Because testosterone changes with age, increasing during adolescence and decreasing slightly through adulthood, we used *gamlss* [52] to fit models to log testosterone based on log age and, in some models, time-since-waking (Fig. 1). Log testosterone was used due to roughly log-normal distributions and the fact that most hormones and other biomarkers are log distributed and likely have log-scale physiological effects. Time-since-waking was entered as a linear variable in minutes. These models were used to generate age and time-since-waking-corrected testosterone z-scores. Height and BMI were converted to Shuar specific age-corrected z-scores using the *localgrowth* package (https://github.com/adblackwell/localgrowth) based on references in Urlacher et al. [42].

We used the *brms* package to fit Bayesian models via Stan [53]. Due to the imputation of missing heights and weights, models were fit to all 10 imputed datasets and the posteriors pooled. Each model was run with three chains, a warmup of 2000 iterations and a sampling period of 2000 post-warmup, for a total of 60 000 samples per model. All models were fit with non-informative normalizing priors. For models with parasite egg counts as the dependent variable, we fit hurdle models that include a hurdle component, which models the probability of obtaining a zero (i.e. being uninfected) and a continuous component, modelled with a log-normal distribution to fit the distribution of egg count values. For ease of interpretability, we report hurdle parameters as the inverse of *brms* output so that they indicate odds of infection (rather than odds of no infection).

Hurdle models include a single mean age and time-since-waking corrected z-score for testosterone per individual (i.e. testosterone concentration averaged across samples for that individual, with each testosterone sample corrected for time-since-waking, and the overall average value corrected for participant age). As a robustness check, we also modeled age-corrected testosterone as a function of infection status (see Supplementary Information). These models use testosterone z-scores corrected only for age, since sampling time is included as a variable in the models. We also fit separate hurdle models for morning and evening testosterone z-scores to compare to the combined models. To examine hormone and infection patterns across life stages, analyses considered adolescents (under 20 years old) and adults (20 years old and older) separately, as well as pooled together, and use age and sample time corrected testosterone z-scores instead of raw testosterone values.

## RESULTS

**Figure 2:**
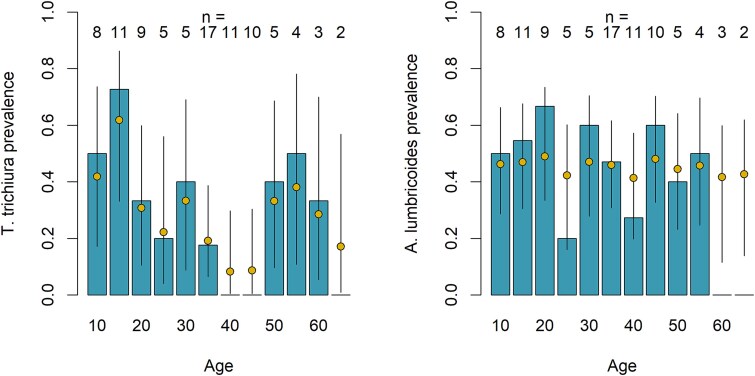
Infection prevalence by age group. Bars show the empirically observed prevalence. Lines and dots show the median and 95% posterior predicted prevalence from logistic models with age category as a random effect.

Table 1 presents frequencies and average values of analysis variables. Overall, 19 adolescents (mean age 15.9 years, range 12.5–19.7) and 71 adults (mean age 40.8 years, range 20–67.7) were included in analyses. Consistent with general male developmental patterns, testosterone levels increased throughout adolescence, peaked in the early 20s, and were slightly lower among older adult male participants (Fig. 1). Among adolescents, testosterone levels were associated with greater height for age (*r* = 0.57, *P* = .02). Overall, 57.8% of participants were infected with at least one parasite species, with 45.6% of participants infected with *A. lumbricoides* and 28.9% with *T. trichiura*; 16.7% were infected with both parasites. Mean untransformed eggs per gram (EPG; indicative of individual parasite load) values were 3070 and 62.7 eggs per gram for *A. lumbricoides* and *T. trichiura*, respectively. Infection prevalence and mean egg count was higher for adolescents than adults (Table 1), and *T. trichiura* prevalence was relatively high in 15–20-year-olds (Fig. 2).

**Table 1:** Sample descriptive statistics by age group.

	**Adolescents (n = 19)**		**Adults (n = 71)**		**All (n = 90)**	
Variable	Missing	Mean (SD)	Missing	Mean (SD)	Missing	Mean (SD)
Age	0	15.9 (2.7)	0	40.8 (11.8)	0	35.5 (14.7)
AM Samples (#)	0	2.7 (0.5)	0	2.7 (0.4)	0	2.7 (0.4)
PM Samples (#)	0	2.7 (0.5)	0	2.6 (0.6)	0	2.6 (0.5)
AM Testosterone (pmol/L)	0	315.2 (139.1)	0	447.0 (194.1)	0	419.2 (191.0)
PM Testosterone (pmol/L)	0	183.8 (93.7)	0	260.6 (92.4)	0	244.4 (97.4)
Testosterone Z	0	−0.2 (0.8)	0	−0.03 (0.8)	0	−0.07 (0.8)
*A. lumbricoides* (Y/N)	0	0.5	0	0.4	0	0.5
*A. lumbricoides* EPG	0	5411.4 (10508.1)	0	2442.6 (4631.5)	0	3069.3 (6378.7)
*T. trichiura* (Y/N)	0	0.6	0	0.2	0	0.3
*T trichiura* EPG	0	224.8 (394.3)	0	19.3 (51.3)	0	62.7 (201.6)
Height (cm)	4	151.1 (10.6)	6	160.0 (4.3)	10	158.4 (6.9)
Weight (kg)	4	48.1 (9.9)	6	64.8 (6.3)	10	61.6 (9.6)
BMI	4	20.8 (1.8)	6	25.3 (2.4)	10	24.5 (2.9)
Height Z	4	−0.01 (1.1)	6	−0.09 (1.1)	10	−0.07 (1.1)
Weight Z	4	−0.04 (1.4)	6	0.4 (1.3)	10	0.3 (1.3)
BMI Z	4	−0.1 (1.3)	6	0.3 (1.3)	10	0.2 (1.3)

**Figure 3:**
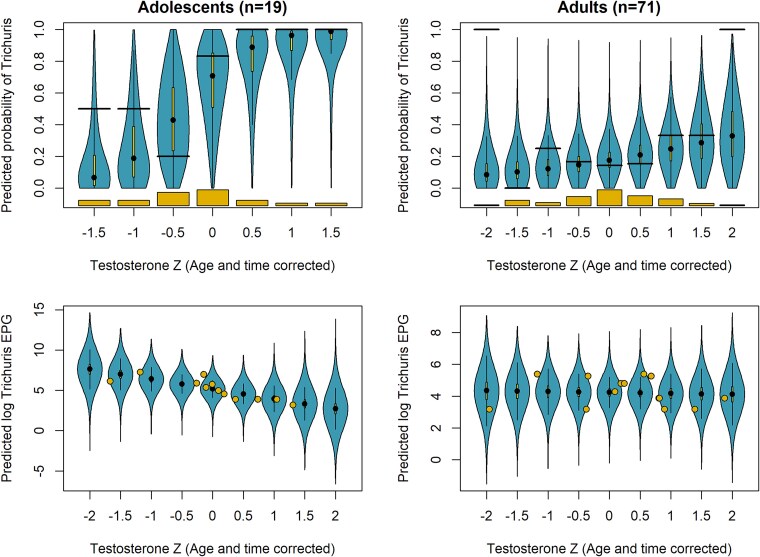
*Trichuris trichiura* infection by testosterone z-score for adolescents (left) and adults (right). Violin plots show the model predicted probability of infection (top) and eggs per gram for those infected (bottom). Horizontal lines on the top figures show actual empirical infection prevalence, yellow dots on the bottom figures show observed eggs-per-gram for infected individuals. Yellow bars at the bottom of the top row show the relative number of observations at each testosterone z-score category.

We fit hurdle models to *T. trichiura* and *A. lumbricoides* EPG to simultaneously model the association between testosterone and infection status (to test prediction 1a) and the association between testosterone and parasite load among infected individuals (to test prediction 2). Given the different testosterone profiles for adolescents and adults, models were divided by age (to test prediction 1b). A combined model was also run. In adolescents, age and sample time corrected testosterone z-scores were associated with an increased odds of *T. trichiura* infection (β = 2.45; OR = 11.59, 95%: 1.16, 188.7; Table 2), though given the small sample size, the 95% posterior credibility interval for the parameter is quite wide. In contrast, higher testosterone was associated with lower *T. trichiura* EPG among adolescents who were infected (β = −1.21; 95% −2.21, −0.10). In adults, parameter estimates were in the same direction, but mean estimates were smaller and the 95% CI intervals did not exclude zero, suggesting little evidence for an association. These associations are illustrated in Fig. 3. For *A. lumbricoides* there was little evidence for associations between testosterone and odds of infection or infection intensity at any age (Table S1**;**  Fig. S1).

**Table 2:** *Trichuris trichiura* infection hurdle models by age group. Values are mean and 95% posterior probability intervals.

**Independent**	**Adolescents**	**Adults**	**All**
HU: Intercept	−2.14 (−17.73,10.87)	−0.64 (−3.16,1.98)	0.99 (−1.09, 3.09)
HU: Testosterone Z	2.45 (0.15, 5.24)	0.41 (−0.44,1.29)	0.47 (−0.25, 1.23)
HU: Age (years)	0.19 (−0.59, 1.15)	−0.02 (−0.08,0.03)	−0.06 (−0.11,-0.02)
HU: SD (Community)	5.14 (1.15,13.05)	1.24 (0.07,3.30)	2.07 (0.72, 4.38)
EPG: Intercept	3.44 (−1.43, 8.00)	4.40 (2.25,6.44)	5.45 (4.46, 6.47)
EPG: Testosterone Z	−1.21 (−2.21,-0.10)	−0.05 (−0.76,0.71)	−0.60 (−1.07,-0.12)
EPG: Age (years)	0.11 (−0.18, 0.41)	0.00 (−0.05,0.04)	−0.03 (−0.05, 0.00)
EPG: SD (Community)	0.75 (0.02, 3.04)	0.51 (0.02,1.76)	0.35 (0.01, 1.21)

HU = hurdle component; coded to reflect odds of exceeding zero infection; parameters reflect the log odds. EPG = eggs per gram component, for those with EPG greater than zero; parameters are in units of log outcome. ‘SD’ indicates the standard deviation of a random effect term.

## CONCLUSIONS

Overall, this study suggests that testosterone exhibits complex relationships with age and different parasite infection types. In adolescents, testosterone levels were positively related to *T. trichiura* infection risk and negatively associated with parasite load. This suggests that, in line with the ICHH [12], higher testosterone levels may be linked with an increased initial risk of infection, but downregulated as infection intensity rises, However, these patterns were only clearly apparent for adolescents, not adults, and only in the case of *T. trichiura,* and not *A. lumbricoides* infection. These results align with recent work showing no significant relationship between testosterone and oxidative stress in adult Shuar males [54]. Oxidative stress from maintaining testosterone-dependent traits has been proposed as a pathway contributing to disease, and animal studies link elevated testosterone with oxidative damage and parasite infection risk [55]. It is therefore notable that this same relationship was not observed among Shuar men, suggesting that testosterone may not be linked with oxidative stress and associated parasitic disease risk during adulthood in this sample, further highlighting the complex context-dependent associations evident between testosterone and immune function.

### Testosterone, parasitic infection patterns, and host developmental stage

Higher testosterone levels among Shuar adolescents were associated with increased odds of *T. trichiura* infection. Specifically, infection peaked in the 15–20 year-old participants, coinciding with increasing testosterone levels and testosterone-linked developmental patterns (i.e. increased height for age in this sample). This finding is consistent with other work demonstrating that rising testosterone levels during adolescence lead to significant increases in bone and muscle growth [56, 57]. It therefore seems likely that rising testosterone levels during this period increase the odds of *T. trichiura* infection—perhaps because energetic resources are shifted from immune activity to physical growth and reproductive effort. This suggests that increased infection risk at this age might be linked with hormonal changes. These findings are consistent with previous research suggesting that males are more susceptible than females to certain types of parasitic infection (e.g. Lymphatic Filariasis and Leishmaniasis) after puberty, a pattern hypothesized to be linked with changes in androgen levels [33, 34].

These results suggest that testosterone inhibits aspects of immune function that may increase preliminary infection risk. Previous work suggests that testosterone may dampen key antibody responses to infection, reduce vaccination effectiveness, and suppress T and B cell development and activity in humans [58]. Energetic tradeoffs drive this relationship, such that the energetic costs associated with high testosterone levels (e.g. the development and maintenance of masculine secondary sexual characteristics) necessitate tradeoffs with other energetically expensive biological functions, including immune activity. However, our results demonstrate that after initial infection, *T. trichiura* EPG (a proxy measure of infection intensity and duration) exhibits an inverse relationship with testosterone levels among adolescents. These findings might indicate that heavier infections necessitate increased energetic investment in immune activity to prevent more severe symptoms during key developmental periods [59, 60], leading to lower testosterone levels until pathogen burden is reduced. Interestingly, the associations between *T. trichiura* infection load and testosterone levels are no longer apparent after adolescence, suggesting that tradeoffs between infection-related immune activity and other biological functions may be attenuated after adult stature is achieved.

Growing evidence suggests that hosts may shift from resisting to tolerating parasites when the costs of mounting a full immune response—including tradeoffs with other physiological processes and immunopathology from prolonged infection—outweigh the benefits of complete parasite clearance [59]. It is possible that testosterone plays a role in this process. Many types of immune cells (e.g. cells in the thymus, spleen, and bone marrow) have hormone receptors, including those specific to testosterone [61]. Testosterone has been linked with suppressed Th2 cell differentiation—the class of helper T cells generally linked with anti-inflammatory cytokine production and the generation of humoral immune responses to helminth infection. This Th2 suppression can inhibit intestinal helminth expulsion and prevent infection clearance [58, 62]. At the same time, testosterone may induce the production of regulatory T cells that moderate other immune responses favoring infection resistance [58, 63]. Testosterone may also increase production of key cytokines (e.g. IL-10) known to regulate the activity of other immune cells (e.g. dendritic cells) [58, 64]. Thus, testosterone appears to dampen certain aspects of immune activity, while enhancing others. Testosterone-linked immunomodulatory effects may cumulatively promote host infection tolerance during low intensity infections, contributing to the inverse relationship between testosterone levels and *T. trichiura* infection load documented here.

Behavioral changes during adolescence may also help explain these patterns. In adolescence, shifts in travel, social interactions, and subsistence activities and responsibilities have been documented in other subsistence-level populations [65]. Testosterone may contribute to these behavioral changes by influencing physiological processes and increasing investment in reproductive effort, both of which occur alongside shifts in social roles and expectations. For example, adolescent boys in subsistence-level populations play a larger role in food production compared to age-matched girls [66] which may expose adolescent boys to more sources of parasitic infection (i.e. environmentally transmitted pathogens such as *T. trichiura*).

### Testosterone levels in relation to parasite species

Testosterone levels were largely unrelated to variation in *A. lumbricoides* infection risk or EPG values. This may be due to consistently high rates of *A. lumbricoides* exposure and infection in this population [30–32], and co-evolution of mechanisms that promote tolerance rather than full parasite clearance in these conditions. *Ascaris lumbricoides* is characterized by prolific egg production and infection at relatively low levels of exposure compared to other helminth species [67]. Thus, study participants of all ages face chronic (re)infection and likely developed immune responses that tolerate some level of *A. lumbricoides* infection, independent of reproductive hormone levels. This finding aligns with previous work among Indigenous Tsimane, which documented significant associations between hookworm infection and pregnancy status, while *A. lumbricoides* infections did not fluctuate during pregnancy [68]. These results suggest that reproductive hormone levels are linked with variable infection patterns for some STH species, but not for ubiquitous *A. lumbricoides*.

Additionally, differences in parasite life cycles and behaviors can affect tradeoffs between host reproductive function and immune activity, affecting testosterone levels. Specifically, STH infections such as *A. lumbricoides* (which feeds through passive nutrient absorption) typically elicit a Th2 biased immune response. However, *T. trichiura* infections appear to trigger a stronger Th1 immune response (i.e. immune activity linked with the production of pro-inflammatory cytokines and largely reliant on the action of neutrophils) or a mixed Th1/Th2 response, likely due to host tissue damage caused by direct parasite attachment to the intestinal walls [69, 70]. *Ascaris lumbricoides* infections may therefore result in less costly immune responses, while heavier *T. trichiura* infections may lead to more acute and energetically expensive inflammatory immune activity that necessitates greater physiological tradeoffs, resulting in lower testosterone levels during infection. Variation in parasite transmission routes may also influence these patterns. Previous work among the Shuar suggests that *A. lumbricoides* exposure may be more common around the home (e.g. via contaminated water sources), while *T. trichiura* exposure may be shaped more strongly by interactions with contaminated soil and market integration processes (e.g. increased travel) [31, 32]. As noted above, behavioral changes during adolescence may consequently alter individual infection risk, potentially increasing exposure to *T. trichiura* through these pathways.

### Limitations and strengths

The present study has several limitations. First, these analyses are cross-sectional. Longitudinal data are needed to assess how testosterone shapes disease risk and varies over the course of individual development and the duration of an infection. Additionally, immune activity may vary throughout parasite life cycles, and this variation in immune costs may not be captured by the infection status and intensity measures. Second, the sample was relatively small, limiting our ability to detect small or moderate effects. Third, this study focused on two parasite species common among the Shuar and reliably identified with the Kato Katz method. It is possible that participants were infected with additional parasite species that we were unable to accurately detect with the methods used. The addition of different classes of parasites would clarify how testosterone profiles affect disease risk more broadly. Finally, phenotypic correlation can make it difficult to ascertain the effects of testosterone on infection patterns. Individual energetic budgets could influence disease risk, such that males who have inherently larger energetic budgets can maintain high testosterone levels while still controlling parasitic infections, thereby obscuring hypothesized tradeoffs at the population-level [25]. Yet, phenotypic correlation is thought to be less of an issue in low-resource contexts—such as this study site—where differences in individual resource access are more constrained and tradeoffs between distinct biological functions are expected to be more substantial [25]. Despite these limitations, the collection of morning and evening saliva samples over three consecutive days allowed for a more nuanced examination of testosterone level variation in relation to immune function than is possible with datasets reliant on fewer participant samples. In doing so, this study demonstrates complex relationships between testosterone levels and human helminth infection patterns, highlighting the importance of host life stage, parasite species, and pathogen load.

## CONCLUSIONS

This study showed that among males in a natural fertility population experiencing resource constraints and high pathogen exposure, associations among testosterone profiles and parasite infection measures depend on host life stage, parasite species, and infection intensity. Results show that during adolescence, higher testosterone levels are associated with greater initial infection risk for one parasite species (*T. trichiura*) but not another (*A. lumbricoides*), suggesting high testosterone suppresses some aspects of immune activity during certain developmental stages. However, *T. trichiura* infection intensity was associated with lower testosterone levels, suggesting an increased investment in immune activity during heavy infection and a trade-off between immunity and testosterone-linked biological functions, including growth and reproduction. While some of the patterns were in line with the ICHH, participant age and STH species significantly shaped these associations, demonstrating how host life stage and parasite behavior results in complex testosterone-immunity tradeoffs. Consequently, although the ICHH may provide a useful framework for testing testosterone-immunity tradeoffs, it is insufficient to capture the full range of diverse parasite–host interactions, as well as variation in human immune-related life history tradeoffs across the lifespan.

## Supplementary Material

EMPH_supplementary_clean_eoag013

## Data Availability

The Indigenous Shuar that are the focus of this research have contended with a history of marginalization and ongoing territorial disputes, and conflicts of interest with colonists, the Ecuadorian State, and international corporations. The research team is committed to transparency in research and open access to scientific data, yet ethical considerations prevent us from posting our data publicly to a third-party server. However, the complete de-identified dataset supporting this article will be made available to qualified researchers, clinicians, and others upon request, in accordance with ethical obligations, data use expectations, and data sharing agreements. These are central to the welfare of our Indigenous participants and their agreements to participate in research. We have formalized our data sharing process and have made this process accessible through use of a detailed Data Request Form available on the Shuar Health and Life History Project website (https://www.shuarproject.org/data-sharing). This process upholds our obligations to protect the confidentiality and understandings with study participants, while enabling us to effectively share relevant information with individuals who are qualified to ensure the confidentiality and ethical use of these data. Conduct of this research was approved by the University of Oregon Institutional Review Board (IRB: Protocol Number: #09012010.010, researchcompliance@uoregon.edu), the *Federacion Interprovincial de Centros Shuar* (FICSH), participant community leaders, and community members.
