# Supplementary material for: Amazonian Shuar testosterone levels are related to parasite infection: immunocompetence handicap hypothesis implications
Source: Evol Med Public Health. 2026 Jun 28;14(1):eoag013. doi: 10.1093/emph/eoag013 (PMC13395098; doi:10.1093/emph/eoag013)
Supplement: EMPH_supplementary_clean_eoag013 [file emph_supplementary_clean_eoag013.docx]

**Supplementary Information**

**Household clustering**

Household clustering was negligible in the sample (adolescents: 19 participants across 14 households; adults: 71 participants across 65 households). The limited number of multi-participant households provided insufficient variance to estimate a household-level effect reliably. Household ID was therefore not included as a control variable in the final models.

**Robustness checks**

We performed three robustness checks on study results. First, we included BMI z-scores (BMI-Z) and height z-scores (HeightZ) in the models as phenotypic controls. BMI-Z is a rough control for energetic status, and HeightZ is primarily a proxy for the pace of pubertal development in adolescents. There was little evidence for an association between either of these controls and either infection (**Tables S2, S3**), with posterior estimates encompassing zero. Including these controls did not meaningfully impact mean posterior estimates for the association with testosterone, though they did widen the posterior intervals suggesting caution may be warranted in interpreting these data.

For the second robustness check we reversed the models, making testosterone the dependent variable and infection status and load the independent predictors (**Table S4**). These models have the advantage of using all the testosterone samples collected, rather than average participant testosterone concentration. These models also control for BMIZ and HeightZ. In these models *T. trichiura* infection was related to higher testosterone z-score (β = 1.39; 95%: 0.36, 2.17) but EPG z-score was associated with lower testosterone (β = -0.3; 95%: -0.70, -0.04) for adolescents, consistent with the hurdle models. No other associations with infection were observed.

Finally, we ran hurdle models using only morning or evening age-corrected testosterone z-scores, in order to examine whether morning or evening levels might be more informative (**Tables S5-S8, Fig. S2**). Associations with morning testosterone were similar to those with combined testosterone levels. However, we found that evening testosterone was associated with increased odds of *T. trichiura* infection in both adolescents (β = 2.40; OR = 11.02; 95%: 1.15, 172.43) and adults (β = 0.86; OR = 2.36; 95%: 1.01, 5.87), although the association with EPG in adolescents was less certain than with morning testosterone.

Figures


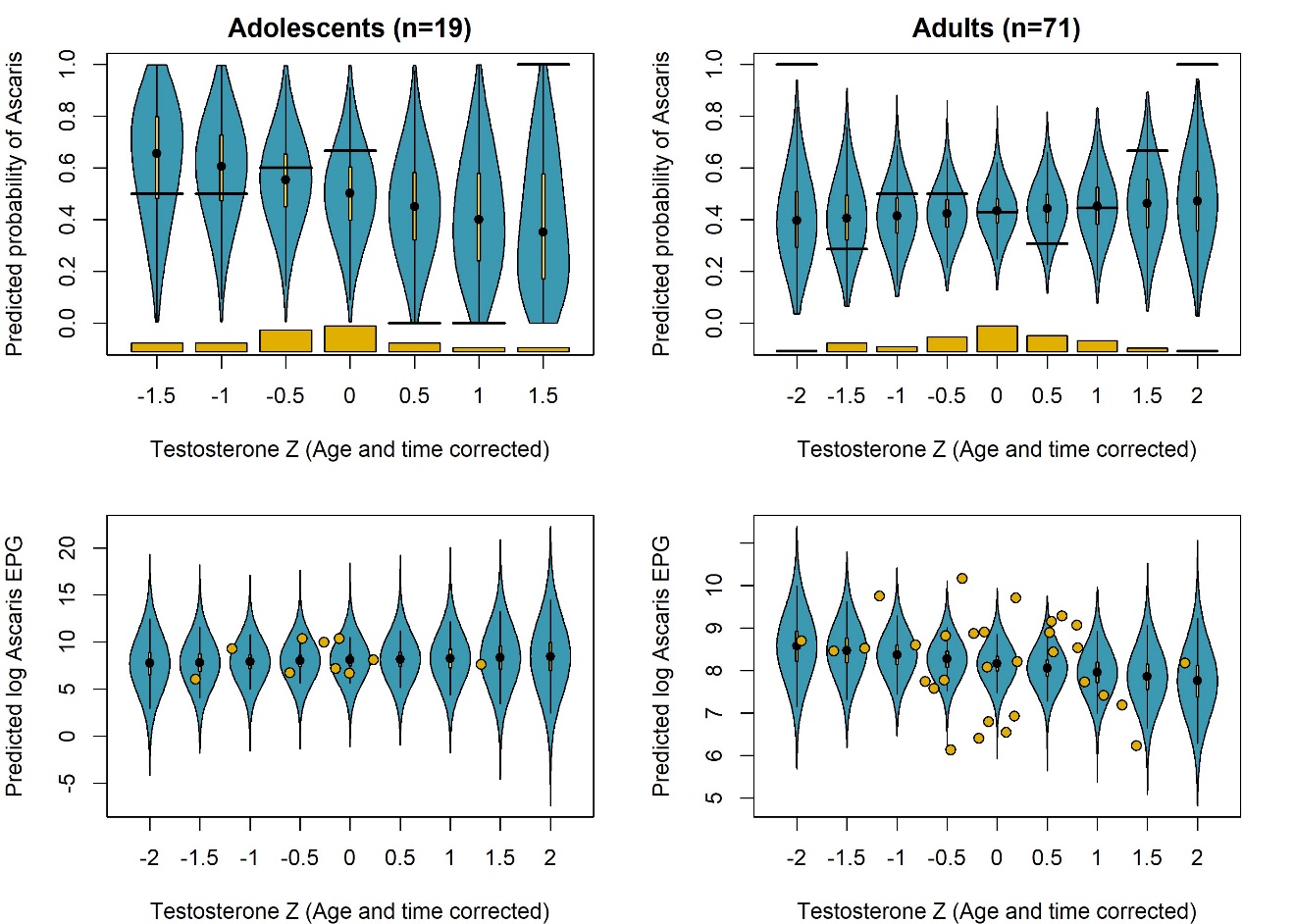


Figure S1. *Ascaris lumbricoides* infection by testosterone z-score for adolescents (left) and adults (right). Violin plots show the model predicted probability of infection (top) and eggs per gram for those infected (bottom). Horizontal lines on the top figures show actual empirical infection prevalence, yellow dots on the bottom figures show observed eggs-per-gram for infected individuals. Yellow bars at the bottom of the top row show the relative number of observations at each testosterone z-score category.


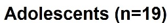

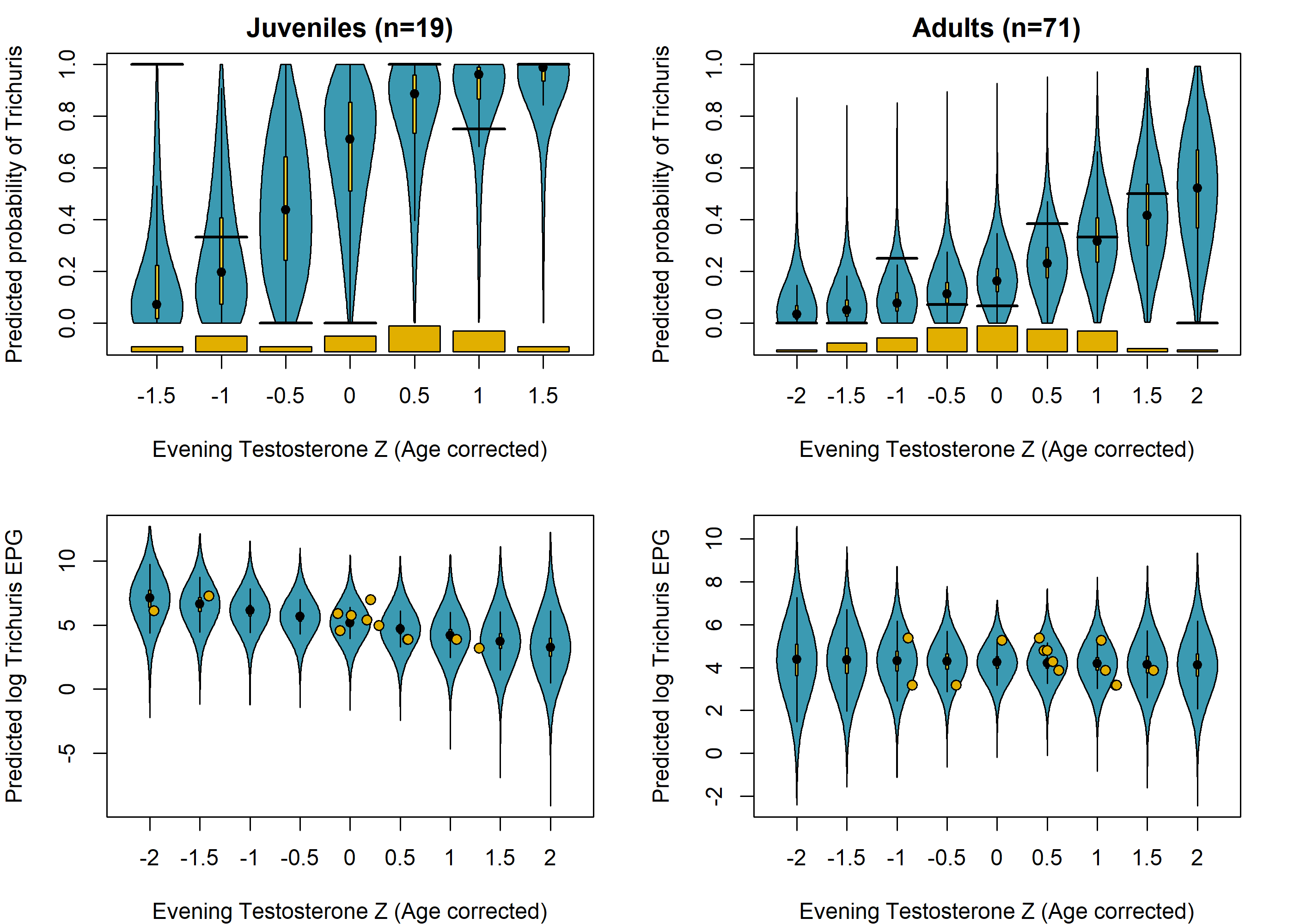


Fig. S2. *Trichuris trichiura* infection by evening testosterone z-score for adolescents (left) and adults (right). Violin plots show the model predicted probability of infection (top) and eggs per gram for those infection (bottom). Horizontal lines on the top figures show actual empirical infection prevalence, yellow dots on the bottom figures show observed eggs-per-gram for infected individuals. Yellow bars at the bottom of the top row show the relative number of observations at each testosterone z-score category.

Tables

**Table S1.** *Ascaris lumbricoides* infection hurdle models. Values are mean and 95% posterior probability intervals. HU=hurdle component; coded to reflect odds of exceeding zero infection; parameters reflect the log odds. EPG = eggs per gram component, for those with EPG greater than zero; parameters are in units of log outcome. 'sd' indicates the standard deviation of a random effect term.

| **Independent** | **Adolescents** | **Adults** | **All** |
| --- | --- | --- | --- |
| HU: Intercept | -4.21 (-11.79, 2.67) | 1.36 (-0.47,3.29) | 0.73 (-0.44,1.96) |
| HU: Testosterone Z | -0.44 ( -1.95, 0.96) | 0.08 (-0.54,0.70) | 0.05 (-0.48,0.58) |
| HU: Age (years) | 0.27 ( -0.16, 0.73) | -0.04 (-0.09,0.00) | -0.03 (-0.06,0.00) |
| HU: sd(Community) | 0.90 ( 0.03, 3.44) | 0.35 ( 0.01,1.13) | 0.28 ( 0.01,0.90) |
| EPG: Intercept | 3.79 ( -5.84,13.29) | 7.87 ( 6.30,9.44) | 7.99 ( 6.87,9.11) |
| EPG: Testosterone Z | 0.20 ( -1.69, 2.08) | -0.20 (-0.68,0.27) | -0.17 (-0.63,0.29) |
| EPG: Age (years) | 0.27 ( -0.30, 0.83) | 0.01 (-0.03,0.05) | 0.00 (-0.03,0.03) |
| EPG: sd(Community) | 1.38 ( 0.04, 4.94) | 0.43 ( 0.02,1.21) | 0.33 ( 0.01,0.99) |

Table S2. *Trichuris trichiura* hurdle models with phenotypic controls.

| **Independent** | **Juveniles** | **Adults** | **All** |
| --- | --- | --- | --- |
| HU: Intercept | -5.54 (-25.57, 9.90) | -0.93 (-3.71,1.85) | 0.99 (-1.29, 3.33) |
| **HU: Testosterone Z** | **2.39 ( -0.20, 5.31)** | **0.45 (-0.46,1.39)** | **0.55 (-0.20, 1.36)** |
| HU: Age (years) | 0.42 ( -0.52, 1.67) | -0.02 (-0.08,0.05) | -0.06 (-0.11,-0.02) |
| HU: BMIZ | 0.83 ( -1.57, 3.57) | -0.26 (-0.88,0.34) | -0.14 (-0.74, 0.46) |
| HU: HeightZ | 0.73 ( -1.92, 3.69) | 0.16 (-0.59,1.00) | 0.41 (-0.23, 1.13) |
| sd(HU: Community) | 5.64 ( 1.45,14.16) | 1.44 ( 0.09,3.70) | 2.40 ( 0.90, 4.92) |
| Intercept | 3.71 ( -2.79, 9.59) | 4.52 ( 2.05,6.78) | 5.64 ( 4.65, 6.67) |
| Testosterone Z | -1.03 ( -2.33, 0.61) | -0.19 (-1.07,0.72) | -0.58 (-1.04,-0.11) |
| Age (years) | 0.09 ( -0.27, 0.49) | -0.01 (-0.06,0.04) | -0.03 (-0.06, 0.00) |
| BMIZ | -0.24 ( -1.17, 0.70) | 0.00 (-0.61,0.58) | -0.04 (-0.40, 0.32) |
| HeightZ | -0.05 ( -0.89, 0.79) | -0.41 (-1.46,0.66) | -0.42 (-0.85, 0.01) |
| sd(Community) | 1.02 ( 0.03, 3.68) | 0.69 ( 0.03,2.28) | 0.34 ( 0.01, 1.22) |

Values are mean and 95% posterior probability intervals. HU=hurdle component; coded to reflect odds of exceeding zero infection; parameters reflect the log odds. EPG = eggs per gram component, for those with EPG greater than zero; parameters are in units of log outcome. 'sd' indicates the standard deviation of a random effect term.

Table S3. *Ascaris lumbricoides* hurdle models with phenotypic controls.

| **Independent** | **Juveniles** | **Adults** | **All** |
| --- | --- | --- | --- |
| HU: Intercept | -3.80 (-13.26, 5.45) | 1.05 (-1.05,3.18) | 0.58 (-0.66,1.85) |
| HU: Testosterone Z | -0.76 ( -2.70, 1.04) | 0.20 (-0.45,0.87) | 0.07 (-0.48,0.63) |
| HU: Age (years) | 0.24 ( -0.33, 0.83) | -0.03 (-0.08,0.02) | -0.02 (-0.05,0.01) |
| HU: BMIZ | -0.71 ( -2.61, 0.82) | 0.11 (-0.33,0.57) | -0.07 (-0.45,0.31) |
| HU: HeightZ | 0.88 ( -0.95, 2.97) | 0.41 (-0.15,1.04) | 0.29 (-0.18,0.79) |
| sd(HU: Community) | 1.17 ( 0.04, 4.25) | 0.43 ( 0.02,1.36) | 0.31 ( 0.01,0.97) |
| Intercept | 1.40 (-10.03,13.78) | 7.76 ( 6.14,9.39) | 7.89 ( 6.77,9.02) |
| Testosterone Z | -0.46 ( -2.88, 2.23) | -0.17 (-0.69,0.35) | -0.14 (-0.60,0.32) |
| Age (years) | 0.41 ( -0.35, 1.10) | 0.01 (-0.03,0.05) | 0.01 (-0.02,0.04) |
| BMIZ | 0.42 ( -1.81, 2.50) | -0.15 (-0.51,0.22) | -0.13 (-0.46,0.21) |
| HeightZ | 0.35 ( -1.49, 2.12) | 0.13 (-0.32,0.59) | 0.21 (-0.16,0.58) |
| sd(Community) | 1.54 ( 0.05, 5.34) | 0.37 ( 0.01,1.10) | 0.29 ( 0.01,0.92) |

Values are mean and 95% posterior probability intervals. HU=hurdle component; coded to reflect odds of exceeding zero infection; parameters reflect the log odds. EPG = eggs per gram component, for those with EPG greater than zero; parameters are in units of log outcome. 'sd' indicates the standard deviation of a random effect term.

**Table S4.** Models with age-corrected testosterone as the dependent variable**.**

| **Independent** | **Juveniles** | **Adults** | **All** |
| --- | --- | --- | --- |
| Intercept | -0.57 (-2.90, 1.54) | -0.24 (-0.95, 0.48) | -0.47 (-0.97, 0.02) |
| *Ascaris Lumbricoides* (Y/N) | 0.01 (-0.50, 0.60) | 0.19 (-0.34, 0.71) | 0.10 (-0.30, 0.51) |
| *Trichuris trichiura* (Y/N) | 1.39 ( 0.36, 2.17) | 0.34 (-0.32, 1.00) | 0.84 ( 0.35, 1.32) |
| Sample Time (12 hours) | -1.05 (-1.22,-0.87) | -1.10 (-1.23,-0.97) | -1.09 (-1.20,-0.98) |
| Age (years) | 0.00 (-0.14, 0.15) | 0.00 (-0.01, 0.02) | 0.01 ( 0.00, 0.02) |
| BMIZ | -0.06 (-0.39, 0.34) | -0.08 (-0.21, 0.06) | -0.05 (-0.16, 0.07) |
| HeightZ | 0.06 (-0.30, 0.42) | -0.13 (-0.30, 0.05) | -0.06 (-0.20, 0.09) |
| *Ascaris* EPG Z (Infected) | 0.07 (-0.22, 0.30) | -0.14 (-0.45, 0.16) | -0.07 (-0.28, 0.15) |
| *Trichuris* EPG Z (Infected) | -0.38 (-0.70,-0.04) | 0.04 (-0.45, 0.53) | -0.29 (-0.56,-0.02) |
| sd(Community) | 0.55 ( 0.02, 1.60) | 0.18 ( 0.01, 0.52) | 0.12 ( 0.00, 0.37) |
| sd(Individual) | 0.28 ( 0.01, 0.65) | 0.65 ( 0.53, 0.80) | 0.63 ( 0.53, 0.76) |
| sd(Individual Time) | 0.12 ( 0.01, 0.34) | 0.30 ( 0.11, 0.48) | 0.25 ( 0.10, 0.41) |
| cor(Individual,Time) | 0.11 (-0.93, 0.97) | -0.63 (-0.97,-0.13) | -0.68 (-0.98,-0.22) |

Values are mean and 95% posterior probability intervals. 'sd' indicates the standard deviation of a random effect term.

**Table S5.** *Trichuris trichiura* hurdle models with morning testosterone.

| **Independent** | **Juveniles** | **Adults** | **All** |
| --- | --- | --- | --- |
| HU: Intercept | -3.24 (-18.08, 8.83) | -0.45 (-3.03,2.21) | 0.84 (-1.23, 2.97) |
| HU: Morning TZ | 2.30 ( 0.03, 5.04) | -0.01 (-0.74,0.71) | 0.06 (-0.57, 0.70) |
| HU: Age (years) | 0.26 ( -0.47, 1.17) | -0.03 (-0.09,0.03) | -0.06 (-0.11,-0.02) |
| HU: sd(Community) | 5.08 ( 1.24,12.88) | 1.45 ( 0.12,3.60) | 2.17 ( 0.79, 4.54) |
| EPG: Intercept | 4.36 ( -0.26, 8.73) | 4.44 ( 2.31,6.48) | 5.53 ( 4.51, 6.58) |
| EPG: Morning TZ | -1.34 ( -2.28,-0.36) | -0.07 (-0.67,0.58) | -0.52 (-0.95,-0.10) |
| EPG: Age (years) | 0.05 ( -0.23, 0.33) | -0.01 (-0.05,0.04) | -0.03 (-0.06, 0.00) |
| EPG: sd(Community) | 0.75 ( 0.02, 2.84) | 0.51 ( 0.02,1.78) | 0.38 ( 0.01, 1.30) |

Values are mean and 95% posterior probability intervals. HU=hurdle component; coded to reflect odds of exceeding zero infection; parameters reflect the log odds. EPG = eggs per gram component, for those with EPG greater than zero; parameters are in units of log outcome. 'sd' indicates the standard deviation of a random effect term.

**Table S6.** *Trichuris trichiura* hurdle models with evening testosterone**.**

| **Independent** | **Juveniles** | **Adults** | **All** |
| --- | --- | --- | --- |
| HU: Intercept | -1.35 (-17.22,12.45) | -0.52 (-3.05,2.05) | 1.38 (-0.74, 3.65) |
| HU: Evening TZ | 2.40 ( 0.14, 5.15) | 0.86 ( 0.01,1.77) | 0.89 ( 0.15, 1.69) |
| HU: Age (years) | 0.14 ( -0.69, 1.12) | -0.03 (-0.09,0.03) | -0.08 (-0.13,-0.03) |
| HU: sd(Community) | 5.31 ( 1.17,13.61) | 1.11 ( 0.06,3.05) | 2.04 ( 0.67, 4.39) |
| EPG: Intercept | 2.28 ( -3.31, 7.18) | 4.40 ( 2.35,6.36) | 5.32 ( 4.32, 6.31) |
| EPG: Evening TZ | -0.90 ( -1.86, 0.49) | -0.05 (-0.92,0.88) | -0.67 (-1.19,-0.15) |
| EPG: Age (years) | 0.18 ( -0.12, 0.52) | 0.00 (-0.05,0.04) | -0.02 (-0.05, 0.01) |
| EPG: sd(Community) | 0.86 ( 0.02, 3.44) | 0.49 ( 0.02,1.71) | 0.32 ( 0.01, 1.16) |

Values are mean and 95% posterior probability intervals. HU=hurdle component; coded to reflect odds of exceeding zero infection; parameters reflect the log odds. EPG = eggs per gram component, for those with EPG greater than zero; parameters are in units of log outcome. 'sd' indicates the standard deviation of a random effect term.

**Table S7.** *Ascaris lumbricoides* hurdle models with morning testosterone.

| **Independent** | **Juveniles** | **Adults** | **All** |
| --- | --- | --- | --- |
| HU: Intercept | -4.03 (-11.40, 2.77) | 1.33 (-0.55,3.31) | 0.72 (-0.44,1.92) |
| HU: Morning TZ | -0.67 ( -2.24, 0.74) | 0.11 (-0.43,0.65) | 0.05 (-0.42,0.53) |
| HU: Age (years) | 0.25 ( -0.17, 0.71) | -0.04 (-0.09,0.01) | -0.03 (-0.06,0.00) |
| HU: sd(Community) | 0.94 ( 0.03, 3.63) | 0.35 ( 0.01,1.11) | 0.28 ( 0.01,0.89) |
| EPG: Intercept | 3.74 ( -5.65,13.08) | 7.92 ( 6.30,9.55) | 8.02 ( 6.91,9.14) |
| EPG: Morning TZ | 0.33 ( -1.49, 2.10) | -0.15 (-0.53,0.23) | -0.13 (-0.51,0.25) |
| EPG: Age (years) | 0.27 ( -0.29, 0.83) | 0.01 (-0.03,0.05) | 0.00 (-0.03,0.03) |
| EPG: sd(Community) | 1.38 ( 0.05, 4.88) | 0.42 ( 0.02,1.20) | 0.32 ( 0.01,1.00) |

Values are mean and 95% posterior probability intervals. HU=hurdle component; coded to reflect odds of exceeding zero infection; parameters reflect the log odds. EPG = eggs per gram component, for those with EPG greater than zero; parameters are in units of log outcome. 'sd' indicates the standard deviation of a random effect term.

**Table S8.** *Ascaris lumbricoides* hurdle models with evening testosterone**.**

| **Independent** | **Juveniles** | **Adults** | **All** |
| --- | --- | --- | --- |
| HU: Intercept | -4.03 (-11.52, 2.80) | 1.38 (-0.46,3.34) | 0.75 (-0.42,1.98) |
| HU: Evening TZ | -0.14 ( -1.48, 1.18) | 0.06 (-0.55,0.67) | 0.08 (-0.45,0.60) |
| HU: Age (years) | 0.26 ( -0.16, 0.72) | -0.04 (-0.09,0.00) | -0.03 (-0.06,0.00) |
| HU: sd(Community) | 0.89 ( 0.03, 3.35) | 0.35 ( 0.01,1.12) | 0.28 ( 0.01,0.90) |
| EPG: Intercept | 3.73 ( -6.42,13.72) | 7.73 ( 6.16,9.30) | 7.94 ( 6.81,9.08) |
| EPG: Evening TZ | 0.07 ( -1.80, 2.00) | -0.23 (-0.72,0.27) | -0.16 (-0.65,0.32) |
| EPG: Age (years) | 0.27 ( -0.32, 0.86) | 0.01 (-0.03,0.05) | 0.01 (-0.02,0.04) |
| EPG: sd(Community) | 1.35 ( 0.04, 4.85) | 0.44 ( 0.02,1.23) | 0.34 ( 0.01,1.01) |

Values are mean and 95% posterior probability intervals. HU=hurdle component; coded to reflect odds of exceeding zero infection; parameters reflect the log odds. EPG = eggs per gram component, for those with EPG greater than zero; parameters are in units of log outcome. 'sd' indicates the standard deviation of a random effect term.
